# Recurrence of Complement Factor H-Related Protein 5 Nephropathy in a Renal Transplant

**DOI:** 10.1111/j.1600-6143.2010.03333.x

**Published:** 2011-01

**Authors:** K A Vernon, D P Gale, E Goicoechea de Jorge, A G McLean, J Galliford, A Pierides, P H Maxwell, D Taube, M C Pickering, H T Cook

**Affiliations:** a Centre for Complement and Inflammation Research (CCIR), Division of Immunology and Inflammation, Faculty of Medicine, Imperial CollegeLondon, UK; b Imperial College Kidney and Transplant Institute, Imperial CollegeLondon, UK; c Department of Nephrology, Hippocrateon HospitalNicosia, Cyprus; d Division of Medicine, University CollegeLondon, UK

**Keywords:** CFHR5 nephropathy, complement, recurrence, renal transplant

## Abstract

Complement factor H-related protein 5 (CFHR5) nephropathy is a familial renal disease endemic in Cyprus. It is characterized by persistent microscopic hematuria, synpharyngitic macroscopic hematuria and progressive renal impairment. Isolated glomerular accumulation of complement component 3 (C3) is typical with variable degrees of glomerular inflammation. Affected individuals have a heterozygous internal duplication in the *CFHR5* gene, although the mechanism through which this mutation results in renal disease is not understood. Notably, the risk of progressive renal failure in this condition is higher in males than females. We report the first documented case of recurrence of CFHR5 nephropathy in a renal transplant in a 53-year-old Cypriot male. Strikingly, histological changes of CFHR5 nephropathy were evident in the donor kidney 46 days post-transplantation. This unique case demonstrates that renal-derived CFHR5 protein cannot prevent the development of CFHR5 nephropathy.

## Introduction

Complement dysregulation is associated with several distinct patterns of glomerular pathology. Common to glomerular abnormalities associated with defective control of the alternative pathway is deposition of C3 in the absence of significant immunoglobulin (1,2). This pathological appearance typifies a number of conditions associated with genetic or acquired complement dysregulation, including dense deposit disease, C3 glomerulonephritis (C3GN) and CFHR5 nephropathy. ‘C3 glomerulopathy’ has recently been proposed as a new term under which this heterogeneous group of disorders can be classified (1).

C3GN is a feature of CFHR5 nephropathy, a familial renal disease characterized by persistent microscopic hematuria, synpharyngitic macroscopic hematuria and progressive renal failure (3). C3GN may be associated with membranoproliferative or mesangial proliferative features. Endemic in Cyprus, affected individuals have a heterozygous internal duplication in the *CFHR5* gene. Previously, mutations in complement factor H, CD46 (membrane cofactor protein) and factor I were identified among patients with biopsy-proven C3GN (2), but CFHR5 nephropathy is the first description of C3GN associated with a mutation in the *CFHR5* gene. CFHR5 is a member of the complement factor H (CFH) family, a group of highly related proteins encoded by genes located within the regulator of complement activation (RCA) gene cluster on chromosome 1. Comprising CFH, CFH-like protein (CFHL-1) and complement factor H-related proteins 1–5, the proteins are composed of individual domains termed short consensus repeats (SCRs), which display varying degrees of amino acid sequence similarity to each other. CFHR5 is a 65 kDa protein composed of nine SCRs, and the internal duplication in exons 2 and 3 characteristic of CFHR5 nephropathy results in an expressed protein with duplicated SCRs 1 and 2 respectively. Although the role of CFHR5 is not yet fully understood, its complement regulatory activity *in vitro* (4) and co-localization with renal complement deposits *in vivo* (5) suggest that it may play a role in complement regulation within the kidney. Furthermore, the mutant protein has been shown to have reduced affinity for glomerular-bound complement, raising the possibility of impaired targeting to complement within the kidney (3). We report the case of a 53-year-old gentleman with end-stage renal failure (ESRF) secondary to CFHR5 nephropathy, who underwent renal transplantation from a deceased donor and was found to have evidence of disease recurrence in a transplant biopsy 46 days later.

## Case Presentation

**Figure 1:**
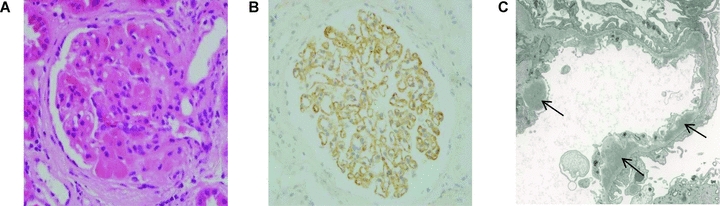
**Native renal biopsy**. (A) Light microscopic appearance showing segmental glomerulosclerosis and hyalinosis and increased mesangial matrix (hematoxylin and eosin). (B) Immunoperoxidase staining showing granular capillary wall C3 deposition. C3 was detected using a polyclonal rabbit anti-human C3c antibody (Dako Ltd., Ely, United Kingdom). (C) Electron micrograph showing multiple subendothelial electron dense deposits (denoted by arrows).

A previously healthy British male with Cypriot ancestry was referred at the age of 36 with persistent microscopic hematuria, episodes of macroscopic hematuria coinciding with upper respiratory tract symptoms, and renal impairment (serum creatinine 178 μmol/L). He was not aware of any family history of renal disease at that time although relatives with CFHR5 nephropathy have subsequently been identified. Physical examination and blood pressure were normal. However urinalysis demonstrated 1+ blood and 1+ protein, and a renal biopsy was performed. Light microscopy revealed 30% glomerular obsolescence with a fibrous crescent in one glomerulus, and a few tubular red cell casts. Immunoperoxidase staining showed capillary wall C3 but notably was negative for IgA. Subendothelial electron dense deposits and rare subepithelial ‘humps’ were seen on electron microscopy (EM), as were mesangial deposits associated with an increase in mesangial cells and matrix. A further decline in renal function 6 years later led to a second biopsy (Figure 1). This demonstrated large segmental scars, capillary wall thickening with double contours and mesangial cell interposition. Granular capillary wall C3 was again evident in the absence of immunoglobulin staining. On EM there were subendothelial and mesangial deposits, and rare subepithelial deposits. These histological features are consistent with C3 glomerulonephritis.

**Figure 2:**
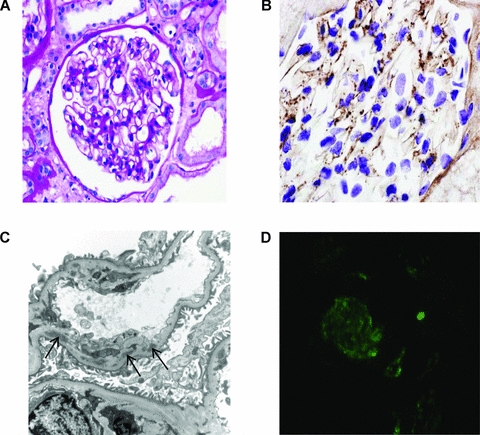
**Renal transplant biopsy**. (A) Light microscopy showing a normal glomerulus (periodic acid-Schiff). (B) Immunoperoxidase staining showing granular capillary wall and mesangial C3 deposition. C3 was detected using a polyclonal rabbit anti-human C3c antibody (Dako Ltd., Ely, United Kingdom). (C) Electron micrograph showing subendothelial deposits (denoted by arrows) with new basement membrane beneath. (D) Glomerular C5b-9 by indirect immunofluorescence. C5b-9 was detected using a monoclonal mouse anti-human C5b-9 antibody (DakoCytomation Ltd., Ely, United Kingdom) and a fluorescein isothiocyanate-labeled goat anti-mouse IgG (Sigma-Aldrich Company Ltd., Dorset, United Kingdom).

Over the following 4 years he suffered progressive renal impairment and required renal replacement therapy at the age of 47. Six years after commencing hemodialysis, during which he had further episodes of macroscopic hematuria, he received a deceased donor renal transplant. The donor was a 65-year-old female with no significant past medical history, who had a creatinine of 82 μmol/L. HLA matching demonstrated a 2,1,1 mismatch, but there were no recipient class I or II HLA antibodies at the time of transplantation, and this proceeded with no complications. Immunosuppression included alemtuzumab and corticosteroids peri-operatively, with tacrolimus monotherapy continued at discharge. Although his renal function initially improved, his creatinine stabilized at 186 μmol/L, and in the presence of persistent microscopic hematuria and following a single episode of macroscopic hematuria (with a urine protein: creatinine ratio of 56 mg/mmol), he underwent a renal transplant biopsy, 46 days after transplantation (Figure 2).

Light microscopy showed occasional neutrophils in the capillary loops of one glomerulus and a small area of tubulointerstitial fibrosis. Immunoperoxidase staining showed capillary wall granular C3 and complement component 9 (C9), whilst electron microscopy showed increased mesangial matrix associated with scattered mesangial and subendothelial deposits (with new basement membrane beneath in two areas; Figure 2C). Complement component 4d (C4d) staining was negative. The findings in the renal transplant were therefore consistent with a recurrence of his original disease. Serum C3 was 0.73 g/L (normal range 0.7–1.7 g/L) at the time of biopsy, whilst CFH and factor I levels were 320 mg/L (207% of normal control) and 32 mg/L (177% of normal control), respectively.

**Figure 3:**
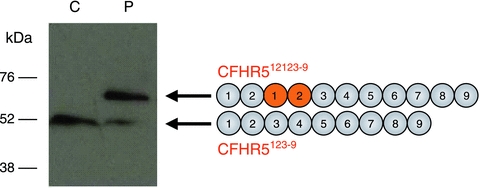
**Internal duplication of CFHR5. Western blot of serum for CFHR5 and schematic depicting normal and mutant CFHR5 proteins (denoted CFHR5^123–9^ and CFHR5^12123–9^ respectively)**. A polyclonal anti-CFHR5 antibody was used to detect the wild-type and mutant CFHR5 proteins in sera from the patient (P) and control (C). The wild-type CFHR5 protein consists of nine protein subunits termed short consensus repeat (SCR) domains. The internal duplication of exons 2 and 3 in the mutant *CFHR5* gene results in a mutant protein that contains two additional SCR domains (duplicated protein domains depicted in orange in the schematic). The mutant CFHR5 protein therefore has a higher molecular weight than the wild-type protein. The mutant protein was readily detectable in the patient but not in the control sample. The presence of the wild-type protein in the patient sample confirmed that the mutation is present in heterozygosity in this individual.

A second transplant biopsy performed 3 months later on account of a rise in the serum creatinine to 250 μmol/L, showed granular (mainly mesangial) C3 staining associated with subendothelial, mesangial and subepithelial deposits. Indirect immunofluorescence also showed the presence of glomerular complement components 5b (C5b)-9 (Figure 2D). Polymerase chain reaction (PCR) using genomic DNA isolated from peripheral blood monocytes and serum CFHR5 western blot analysis (Figure 3) revealed the presence of the heterozygous internal duplication in the *CFHR5* gene, confirming the diagnosis of CFHR5 nephropathy.

## Discussion

To our knowledge this is the first description of recurrence of CFHR5 nephropathy in a transplant. The recurrence of CFHR5 nephropathy in an unrelated kidney demonstrates that local synthesis of normal CFHR5 by the kidney is not sufficient to prevent disease. However, we are aware of two other incompletely characterized cases with the CFHR5 mutation and renal disease, in which good allograft function was evident one decade after deceased donor renal transplantation. Firstly, a Cypriot male with a renal biopsy demonstrating C3GN reached ESRF at the age of 46. A deceased donor renal transplant was performed at the age of 48 and he died 12 years later from a myocardial infarction. The serum creatinine 3 months before his death was 89 μmol/L and the graft was never biopsied. He was a member of a family described previously and genotyping of his daughter and the offspring of his cousin demonstrated that he was an obligate carrier of the CFHR5 mutation (individual III-1 from family 1 in reference (3)). Secondly, a 40-year-old Cypriot individual with ESRF and small kidneys, who presented with macroscopic hematuria at the age of ten but had been lost to follow up, received a deceased donor renal transplant at the age of 43. The transplant functioned well for 10 years until it was lost following atheroembolic complications of diagnostic coronary angiography. He returned to hemodialysis and died 6 years later. Subsequent molecular testing on stored genomic DNA demonstrated the presence of the CFHR5 mutation. There was no record of either native or allograft renal biopsy. These cases, together with the observations that the original disease takes many decades to cause ESRF, suggest that graft loss due to recurrence of CFHR5 nephropathy is not inevitable. Clearly larger studies will be required to establish the clinical course of CFHR5 nephropathy in renal transplantation. In summary, we describe the first reported case of recurrence of CFHR5 nephropathy in an unrelated renal transplant. Notably histological recurrence was demonstrable only 46 days after transplantation.

## Abbreviations

C3: complement component 3
C3c: complement component 3c
C4d: complement component 4d
C5b: complement component 5b
C9: complement component 9
CD46: cluster of differentiation 46 (membrane cofactor protein)
CFH: complement factor H
CFHL-1: CFH-like protein
CFHR5: complement factor H-related protein 5
C3GN: C3 glomerulonephritis
EM: electron microscopy
ESRF: end-stage renal failure
μmol: micromole (s)
mg: milligram (s)
mmol: millimole (s)
PCR: polymerase chain reaction
RCA: regulator of complement activation
SCR: short consensus repeat
